# Protein expression of G-protein inwardly rectifying potassium channels (GIRK) in breast cancer cells

**DOI:** 10.1186/1472-6793-6-8

**Published:** 2006-08-31

**Authors:** Madhu S Dhar, Howard K Plummer

**Affiliations:** 1 Molecular Cancer Analysis Laboratory, Department of Pathobiology, and Department of Large Animal Clinical Sciences, College of Veterinary Medicine, University of Tennessee, Knoxville, TN 37996–4542, USA; 2 Molecular Cancer Analysis Laboratory, Department of Pathobiology, College of Veterinary Medicine, University of Tennessee, Knoxville, TN 37996–4542, USA

## Abstract

**Background:**

Previous data from our laboratory has indicated that a functional link exists between the G-protein-coupled inwardly rectifying potassium (GIRK) channel and the beta-adrenergic receptor pathway in breast cancer cell lines, and these pathways were involved in growth regulation of these cells. Alcohol is an established risk factor for breast cancer and has been found to open GIRK. In order to further investigate GIRK channels in breast cancer and possible alteration by ethanol, we identified GIRK channel protein expression in breast cancer cells.

**Results:**

Cell pellets were collected and membrane protein was isolated to determine GIRK protein expression. GIRK protein was also analyzed by immuno-precipitation. GIRK protein was over-expressed in cells by transfection of GIRK plasmids. Gene expression studies were done by real-time RT-PCR. GIRK protein expression was identified in breast cancer cell lines. Expression of GIRK1 at the indicated molecular weight (MW) (62 kDa) was seen in cell lines MDA-MB-453 and ZR-75-1. In addition, GIRK1 expression was seen at a lower MW (40–42 kDa) in MDA-MB-361, MDA-MB-468, MCF-7, ZR-75-1, and MDA-MB-453 cell lines. To prove the lower MW protein was GIRK1, MDA-MB-453 cells were immuno-precipitated. GIRK2 expression was seen in MDA-MB-468, MCF-7, and ZR-75-1 and was variable in MDA-MB-453, while GIRK4 protein expression was seen in all six cell lines tested. This is the first report indicating GIRK protein expression in breast cancer cells. To determine functionality, MDA-MB-453 cells were stimulated with ethanol. Decreased GIRK1 protein expression levels were seen after treatment with 0.12% ethanol in MDA-MB-453 breast cancer cells. Serum-free media decreased GIRK protein expression, possibly due to lack of estrogen in the media. Transfection of GIRK1 or GIRK4 plasmids increased GIRK1 protein expression and decreased gene expression in MDA-MB-453 breast cancer cells.

**Conclusion:**

Our data indicates that functional GIRK channels exist in breast cancer cells that are involved in cellular signaling.

## Background

Breast cancer is a leading cancer in women, accounting for 32% of new cancers. It is also the second leading cause of cancer death for women, and an estimated 211,240 new cases occurred this year in the USA [1]. Approximately 40% of primary human breast cancers tissues have shown expression of mRNA that encodes a G-protein-coupled inwardly rectifying potassium channel 1 (GIRK1), and this expression of GIRK1 was associated with a more aggressive clinical behavior [2]. Previous data from our laboratory has indicated that a functional link exists between the GIRK1 channel and the beta-adrenergic receptor pathway in breast cancer cell lines, and these pathways were involved in growth regulation of these cells [3,4]. The estrogen receptor positive [ER (+)] cell lines MCF-7, MDA-MB-361, and ZR-75-1 and the ER negative (-) cell line MDA-MB-453 expressed mRNA for the GIRK1 channel, while the ER (-) cell lines MDA-MB-468 and MDA-MB-435S did not express GIRK1 [4].

Ethanol has been shown to increase proliferation and cAMP levels in two ER (+) cell lines (MCF-7 & ZR-75-1) but not in ER (-) cell lines (BT-20 & MDA-MB-231) [5]. Although alcohol is an established risk factor for breast cancer [reviewed in [6] &[7]], little is known of its mechanism of action. Ethanol has been found to open G-protein inwardly rectifying potassium channels (GIRK) in both the heart and brain [8], but no effects of ethanol on GIRK channels in breast cancer have been reported in the literature. However, treatment of MCF-7 breast cancer cells with ethanol increased ERK1/2 activities and resulted in subsequent increased cell growth [9].

In order to further investigate GIRK channels in breast cancer and possible stimulation by ethanol, we identified GIRK channel protein expression in breast cancer cells. Previously we had identified GIRK channel mRNA expression in multiple lung cancer cell lines; however GIRK1 protein expression was seen only in a subset of small cell lung cancer cell lines [10]. GIRK1 must assemble with either GIRK2, 3, or 4 to form functional channels because GIRK1 cannot form channels alone [reviewed in [11]]. Therefore, in the present research, we determined that breast cancer cell lines express GIRK1, 2, or 4 and that ethanol alters the expression of the protein for these channels.

## Results

**Figure 1:**
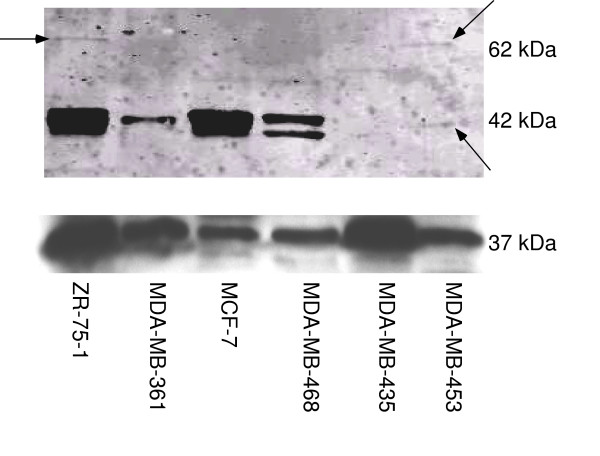
**Protein expression of GIRK1 in six breast cancer cell lines**. Top panel: GIRK1 expression was shown at the correct molecular weight (62 kDa) in ZR-75-1 and MDA-MB-453. GIRK1 expression was also seen at a lower molecular weight (40–42 kDa) for ZR-75-1, MDA-MB-361, MCF-7, MDA-MB-468, and MDA-MB-453. Bottom panel: GAPDH protein was detected in all samples.

**Figure 2:**
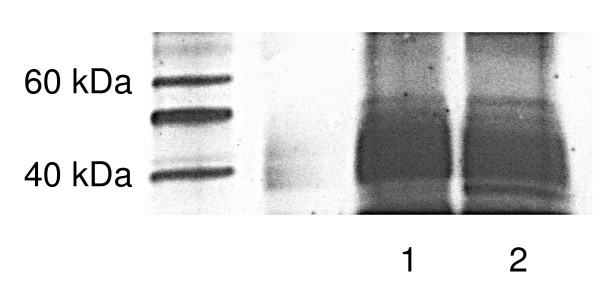
**Immuno-precipitation to determine that the lower MW protein in Figure 1 was GIRK1**. Two samples of MDA-MB-453 cells were immuno-precipitated with goat polyclonal antibody (Santa Cruz) for GIRK1, then separated by Western blot, and probed with rabbit polyclonal antibody used for Figure 1 (Upstate). The highest expression of GIRK1 was at the lower molecular weight in these immuno-precipitated cells. This is a representative gel of two separate experiments.

**Figure 3:**
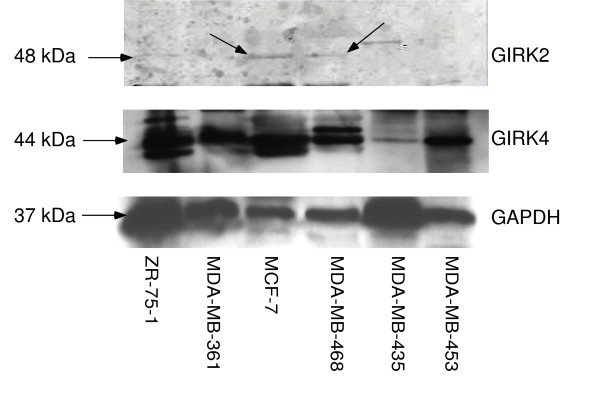
**Protein expression of GIRK2 and GIRK4 in six breast cancer cell lines**. Top panel: GIRK2 expression was shown at the correct molecular weight (48–50 kDa) in ZR-75-1, MCF-7, and MDA-MB-468. Middle panel: GIRK4 expression was shown at the correct molecular weight (44 kDa) in all six cell lines. Bottom panel: GAPDH protein was detected in all samples.

In order to further investigate the effects of GIRK channels in breast cancer, expression of GIRK proteins needed to be determined. Since the predominant GIRK heterotetramers seem to be GIRK1/2 (brain) and GIRK1/4 (cardiac) [reviewed in [11] &[12]], we concentrated on GIRK1, GIRK 2, and GIRK4 expression in these breast cancer cells. GIRK1 expression was shown at the correct molecular weight (62 kDa) in ZR-75-1 and MDA-MB-453 (Figure 1). GIRK1 expression was also seen at a lower molecular weight (40–42 kDa) for ZR-75-1, MDA-MB-361, MCF-7, MDA-MB-468, and MDA-MB-453 (Figure 1). Additional experiments also indicated GIRK1 was also expressed at the lower MW in MDA-MB-453 cells (data not shown). To determine that this lower MW protein was GIRK1 (Figure 1), we immuno-precipitated two samples of MDA-MB-453 with goat polyclonal antibody (Santa Cruz) for GIRK1, then separated by Western blot and probed with rabbit polyclonal antibody (Upstate, Lake Placid, NY). In these immuno-precipitated cells, GIRK1 protein expression was mainly seen at the lower MW (Figure 2). Similar results were seen when MDA-MB-468 cells were immuno-precipitated (data not shown). GIRK1 must assemble with either GIRK2, 3, or 4 to form functional channels because GIRK1 cannot form channels alone [reviewed in [11]]. To determine if the other GIRK channel subunits exist in the breast cancer cells, we identified GIRK2 and GIRK4 expression. GIRK2 expression was shown at the correct molecular weight (48–50 kDa) in ZR-75-1, MCF-7, and MDA-MB-468 (Figure 3). GIRK4 expression was shown at the correct molecular weight (44 kDa) in all six cell lines (Figure 3).

**Figure 4:**
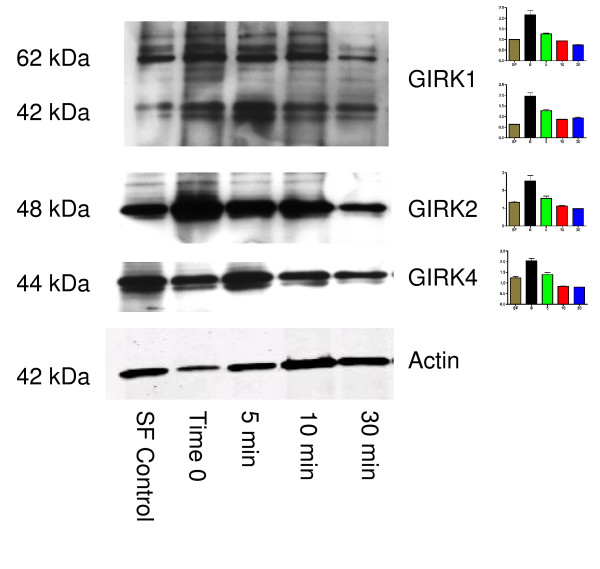
**Decreases in GIRK1, GIRK 2, and GIRK4 protein levels in MDA-MB-453 cells after treatment with ethanol (0.12%) pre-treated with serum free media for 16 hours**. Top panel: GIRK1 protein expression was decreased by 5–30 minute ethanol treatment. GIRK1 expression was changed at both MWs. Serum-free media (used in previous research for 16 hours prior to starting experiment) inhibited GIRK1 protein expression and was not used after these ethanol experiments. Second panel: GIRK2 protein expression was decreased by 5–30 minute ethanol treatments. Serum-free media also inhibited GIRK2 protein expression. Third panel: GIRK4 protein expression was decreased by 5–30 minute ethanol treatment. Serum-free media also decreased GIRK4 protein expression. Bottom panel: Membranes were additionally probed with an antibody for actin to ensure equal loading of protein between samples. This is a representative gel of two separate experiments. Graphs indicate densitometry of bands, and data are shown as a ratio of GIRK/actin.

The majority of our previous research has been done with the ER (-) breast cancer cell line MDA-MB-453 [4]. Therefore, in the present study we continued using this cell line for studies on GIRK channel function. To determine if these GIRK channels are functional, we used ethanol, which is known to open GIRK channels [8]. GIRK1 protein expression was decreased by 5–30 minute ethanol treatment (Figure 4), and GIRK1 expression was changed at both MWs. In addition, both GIRK2 and GIRK4 protein expression were decreased by 5–30 minute ethanol treatment (Figure 4). Data presented in Figure 3 did not indicate expression of GIRK2 in MDA-MB-453 cells. In these experiments with ethanol treatment, however GIRK2 was expressed in MDA-MB-453 cells. Other experiments have indicated variable expression of GIRK2 in MDA-MB-453 cells (data not shown). This data indicates that expression of GIRK channels varies during long-term growth of the cells in culture.

**Figure 5:**
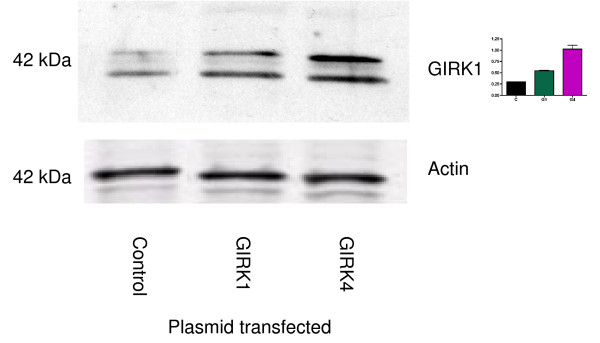
**Western blot indicates an increased GIRK1 protein expression in MDA-MB-453 breast cancer cells 48 hours after transfection with plasmids containing GIRK1 or GIRK4**. Top panel: GIRK1 expression; these bands are at the lower MW (40–42 kDa) indicated in Figure 1. Bottom panel: Membranes were additionally probed with an antibody for actin to ensure equal loading of protein between samples. Graph indicates densitometry of bands, and data are shown as a ratio of GIRK/actin.

To determine the effects of over-expression of GIRK1 and GIRK4 on MDA-MB-453 cells, GIRK1 and GIRK4 plasmids were transfected into MDA-MB-453 cells [13]. Increased GIRK1 protein expression was seen in MDA-MB-453 breast cancer cells 48 hours after transfection with GIRK1 or GIRK4 (Figure 5). These bands are also at the lower MW indicated in Figure 1. On the other hand, 24 hours after transfection of either GIRK1 or GIRK4 plasmids, GIRK1 gene expression was lowered in MDA-MB-453 breast cancer cells. GIRK4 expression was decreased by GIRK1 transfection but increased by GIRK4 transfection. Experiments were performed by real-time RT-PCR and are expressed as gene-specific C_T _value-18S control C_T _value; mean ± standard deviation. Higher C_T _values indicate lower gene expression. GIRK1 gene expression–Control: 16.36 ± 0.53; GIRK1: 18.22 ± 0.52; GIRK4: 18.22 ± 0.12; p < 0.05. GIRK4 gene expression–Control: 19.39 ± 0.61; GIRK1: 21.49 ± 0.02; GIRK4: 16.95 ± 0.78; p < 0.05. All the above data indicate that the lower MW band in these cells is GIRK1.

**Figure 6:**
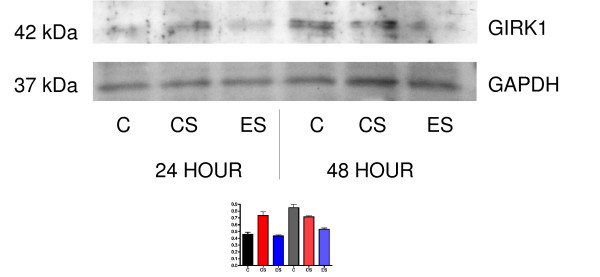
**Western blot indicates changes in GIRK1 protein expression in MDA-MB-453 breast cancer cells 24 and 48 hours after treatment with charcoal-stripped serum ± estradiol. **Top panel: C=control; CS=charcoal-stripped; ES=estradiol. These bands are at the lower MW (40–42 kDa) indicated in Figure 1. At 24 hours, GIRK1 levels were increased by charcoal-stripped serum, and were returned to control levels by estradiol. At 48 hours, GIRK1 levels were unchanged by charcoal-stripped serum, and were decreased by estradiol. Bottom panel: Membranes were additionally probed with an antibody for GAPDH to ensure equal loading of protein between samples. In these experiments, estradiol was dissolved in ethanol (1 mM) and diluted in water/culture media. In the control and charcoal-stripped cultures, 10 μl of 1:100 ethanol:water was added, corresponding to the amount of ethanol added to estradiol treated samples. This is a representative gel of two separate experiments. Graph indicates densitometry of bands, and data are shown as a ratio of GIRK/GAPDH.

We found that the 16-hour serum-free (SF) media pre-treatment normally used for Western blot experiments largely inhibited GIRK1, GIRK2 and GIRK4 protein expression in the MDA-MB-453 cells (Figure 4). Therefore, pre-treatment with SF media was not used in experimental treatments described in Figure 5. This decrease in expression may be due to lack of K^+ ^ions in the media or to removal of estrogen. In support of this hypothesis, treatment of the MDA-MB-453 cell line led to an increase in GIRK1 protein expression after treatment for 24 hours with charcoal-stripped serum, and GIRK1 levels returned to control levels with addition of estradiol (Figure 6). However, we saw a decrease in GIRK protein levels after 48 hours of treatment with estradiol but no change in protein expression in cells treated with charcoal-stripped serum, indicating that charcoal stripping and estradiol have different time-dependent effects on MDA-MB-453 cells (Figure 6). To further investigate this decrease in GIRK1 expression, we incubated MDA-MB-453 for 16 hours (the time that decreased GIRK protein expression) and found this had no effect on GIRK1 mRNA expression (data not shown). However, previous data from our laboratory have indicated that 6–7 days of treatment were needed for β-adrenergic modification of GIRK1 gene expression in the MDA-MB-453 cells [4]. Therefore the MDA-MB-453 cell line was grown in SF media for 7 days, after which real-time PCR revealed decreases in GIRK1 mRNA expression. Data are expressed as described above: Control: 16.76 ± 0.24; SF: 19.12 ± 0.57; p < 0.007. These results indicate that both GIRK1 protein and gene expression are inhibited by serum-free conditions.

## Discussion

Our previous work has indicated that breast cancer cells express mRNA for the GIRK channels. Since protein expression would also be necessary for functional GIRK channels, we determined GIRK protein expression in the six breast cancer cell lines. ZR-75-1, MDA-MB-361, MCF-7 and MDA-MB-453 express GIRK1 mRNA and protein; MDA-MB-435S does not express GIRK1 protein or mRNA; and MDA-MB-468 expresses GIRK1 protein but not mRNA [4]. In addition, data indicated that mRNA for GIRK2 was observed in all cell lines except ZR-75-1 and MDA-MB-435S [4]. GIRK2 protein expression was seen in MDA-MB-468, MCF-7, and ZR-75-1 and has variable expression in MDA-MB-453. Gene expression data also indicated that mRNA for GIRK4 was observed in all cell lines [4], while GIRK4 protein expression was also seen in all six breast cancer cell lines. This is the first report of GIRK protein in breast cancer cells. We previously first reported GIRK1 protein was seen in the three small cell lung cancer (SCLC) cell lines that express GIRK1 mRNA, and determined GIRK1 protein was not expressed in non-SCLC cell lines [10]. The present data also indicates that expression of GIRK channels varies during long-term growth of the cells in culture. This data would correlate with previous data indicating that expression of GIRK1 was associated with a more aggressive clinical behavior [2].

Alcohol is an established risk factor for breast cancer [reviewed in [6] &[7]], but little is known of its mechanism of action. Although treatment of MCF-7 breast cancer cells with ethanol increased ERK1/2 activities and resulted in subsequent increased cell growth [9], no effects of ethanol on GIRK channels in breast cancer have been previously reported in the literature. To determine functionality of the three GIRK channels, MDA-MB-453 cells were treated with ethanol, which has been found to open GIRK channels in both the heart and brain [8]. Since the predominant GIRK heterotetramers seem to be GIRK1/2 and GIRK1/4 [reviewed in [11]], we concentrated on GIRK1, GIRK2, and GIRK4 expression in these cells. GIRK1, GIRK2, and GIRK4 protein expression was decreased by ethanol treatment. We feel that the data indicates that some of the effects of ethanol in breast cancer may be mediated by GIRK channels. Further research is needed to determine whether GIRK1/2 or GIRK1/4 is the predominant heterotetramer in breast cancer. No differences were previously seen between brain-type GIRK1/2 channels and cardiac-type GIRK1/4 channels in their responses to ethanol treatment [8], and these studies involved GIRK1/2 and GIRK1/4 transfected into *Xenopus *oocytes, an artificial system. It is our hypothesis that the predominant heterotetramer in MDA-MB-453 breast cancer cells is GIRK1/4, since we see variable expression of GIRK2 in this cell line.

In our study, GIRK1 was seen at two different molecular weights in breast cancer cell lines. The higher MW (62 kDa) is indicated on the antibody data sheet. In SCLC cell lines, we saw expression only at 62 kDa [10]. We believe that the immuno-precipitation studies likely prove that the lower MW protein is GIRK1. In addition, ethanol decreased GIRK1 expression at both MWs. Furthermore, protein levels of GIRK1 were increased at the lower MW after overexpression of GIRK1 by transfection studies. This data indicates that GIRK1 protein is also expressed at a lower MW in breast cancer cells, a key difference from our data on small cell lung cancer [10]. We believe some of this change in MW may be due to long-term growth of the breast cancer cells in culture also indicating why there is a difference between the MWs in Figure 1 and Figure 5.

It is our contention that the lower MW protein in these gels is GIRK1 and all the data confirm this lower MW. In the research, we used two different GIRK1 antibodies, and the lower MW band appeared in the gels using both antibodies. According to the manufacturer's data sheet (Upstate Biotechnology, Lake Placid, NY) the antibody used in Figure 1 corresponded to amino acids 6–42 of rat GIRK1. As per the manufacturer's data sheet (Santa Cruz, Santa Cruz, CA), the antibody used in Figures 4–6 was raised against a peptide within an internal region of human GIRK1. Since these two antibodies map different regions, we believe it is unlikely that this is an artifact. In addition, previous work from our laboratory indicated the Upstate antibody only appeared at the higher MW in SCLC cell lines [10]. It is also possible that this lower MW was a degradation product of GIRK1, but we think this is unlikely because the same protein samples were used for determining GIRK2 and GIRK4 protein expression, and these samples showed no differences in MW. It is our hypothesis that this lower MW is a splice variant of GIRK1, since splice variants with truncated versions of GIRK1 have been previously reported [14]. Further research is needed in order to confirm this hypothesis.

GIRK1 protein expression was increased by transfection of GIRK1 and GIRK4 plasmids: however, mRNA expression of GIRK1 was decreased by transfection of either GIRK1 or GIRK4. We believe that the increase in GIRK proteins negatively regulates GIRK1 gene expression. It is also our contention that this transfection data confirms previous work indicating GIRK1 is overexpressed in cancer cells [2]. In support of this theory, in the present studies, transfection of GIRK4 leads to an increase in GIRK4 mRNA levels. In addition, SF media decreased GIRK1 expression. We believe that this change is due to lack of estrogen in the media. This is confirmed by our data with charcoal-stripped serum and estradiol. Previous investigators have indicated 17-β-estradiol can modulate GIRK channel activation in the brain, and this modulation is blocked by protein kinase A and protein kinase C inhibitors [15]. It is our contention that effects of some risk factors for breast cancer, such as estrogen and ethanol, can be mediated by GIRK channels.

## Conclusion

Our data indicate that functional GIRK channels that are involved in cellular signaling exist in breast cancer cells. Serum free media (used in previous research involving Western blots for 16 hours prior to starting experiment) inhibited GIRK1 protein expression, possibly due to lack of K^+ ^ions or estrogen. These data indicate that the GIRK channels are functional and an integral part of the intra-cellular signaling in breast cancer.

## Methods

### Cell culture

The human breast cancer cell lines MDA-MB-361, ZR-75-1, MCF-7, MDA-MB-453, MDA-MB-468, and MDA-MB-435S were purchased from the American Type Culture Collection (Rockville, MD). Cells were maintained in RPMI 1640 medium supplemented with fetal bovine serum (10%, v/v), L-glutamine (2 mM), penicillin (100 U/ml), and streptomycin (100 μg/ml) (Mediatech, Herndon, VA) in an environment of 5% CO_2_.

### Chemicals

Estradiol (Sigma St. Louis, MO) was dissolved in ethanol and diluted in water. Ethanol (100%) was purchased from AAPER, Shelbyville, KY.

### Western blots

Cell pellets were collected and membrane protein was isolated as previously described [10]. Additional antibodies for GIRK2 and GIRK4 (Santa Cruz, Santa Cruz, CA) were used in these studies. In some Western blots, membranes were additionally probed with an antibody for actin (Sigma) or GAPDH (Abcam, Cambridge, MA) to ensure equal loading of protein between samples. In the experiment with ethanol treatment (Figure 4) only, cells were exposed to SF media for 16 hours prior to ethanol treatment. In Figure 6, cells were treated with charcoal-stripped serum (Biomeda, Foster City, CA) ± estradiol. Densitometry was done using Scion Image release Alpha 4.0.3.2, available from NIH.

### Transfection studies

We obtained GIRK1 and GIRK4 plasmids (H. Lester, California Institute of Technology) [13] and used Liopofectamine 2000 (Invitrogen, Carlsbad, CA) according to manufacturer instructions to transfect breast cancer cells with 10 μg DNA and 20 μl lipofectamine in a 60 mm dish of cells. Both 24 and 48 hours after transfection, protein or RNA was isolated from cells.

### Real-time RT-PCR

Real-time PCR assays were performed as previously described [4,10].

### Immuno-precipitation

Immuno-precipitation was carried out per the manufacturer's directions (Santa Cruz). Membrane protein (100 μg) extracted from MDA-MB-453 cells was incubated with 300 μl of immuno-precipitation buffer and 3 μg of GIRK1 primary antibody (1 μg/μl) with shaking at 4 degrees for 1 hour. Then 30 μl of protein A/G agarose beads (Santa Cruz) were added and incubated overnight at 4 degrees with constant shaking. The immuno-precipitate was collected by centrifugation at 10,000 rpm for 10 minutes at 4 degrees. Supernatant was carefully aspirated and discarded. The pellet was gently washed 4 times with 1 ml ice cold PBS, each time repeating the centrifugation step above. After the final wash, the supernatant was carefully aspirated and discarded. The immuno-precipitated pellet was resuspended in 21 μl of 3× electrophoresis sample buffer. The samples were boiled for 2 minutes and were subjected to electrophoresis and autoradiography according to standard methods as previously described [10].

## Authors' contributions

MD processed the cells, did immuno-precipitation, and carried out the GIRK western blots. HP conceived the studies, treated and transfected the cells, did the real-time PCR studies, and wrote the manuscript. All authors have read and approved the final manuscript.
